# Supplementary figures and images for: Comparative analysis of piRNA sequences, targets and functions in nematodes
Source: RNA Biol. 2022 Nov 22;19(1):1276–92. doi: 10.1080/15476286.2022.2149170 (PMC9683057; doi:10.1080/15476286.2022.2149170)

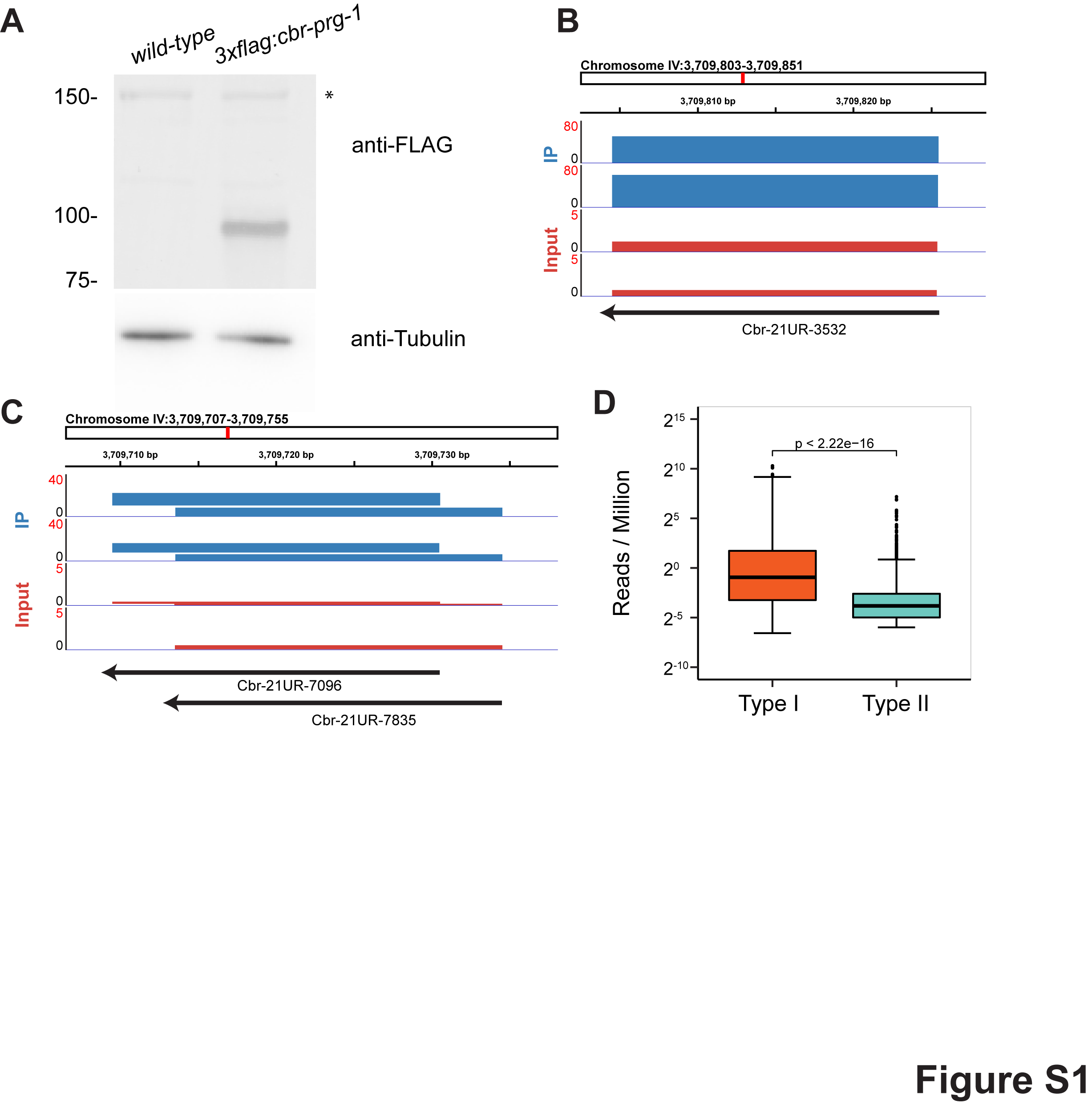

Supplement: Supplemental Material [file KRNB_A_2149170_SM6978.zip › S1.tif]

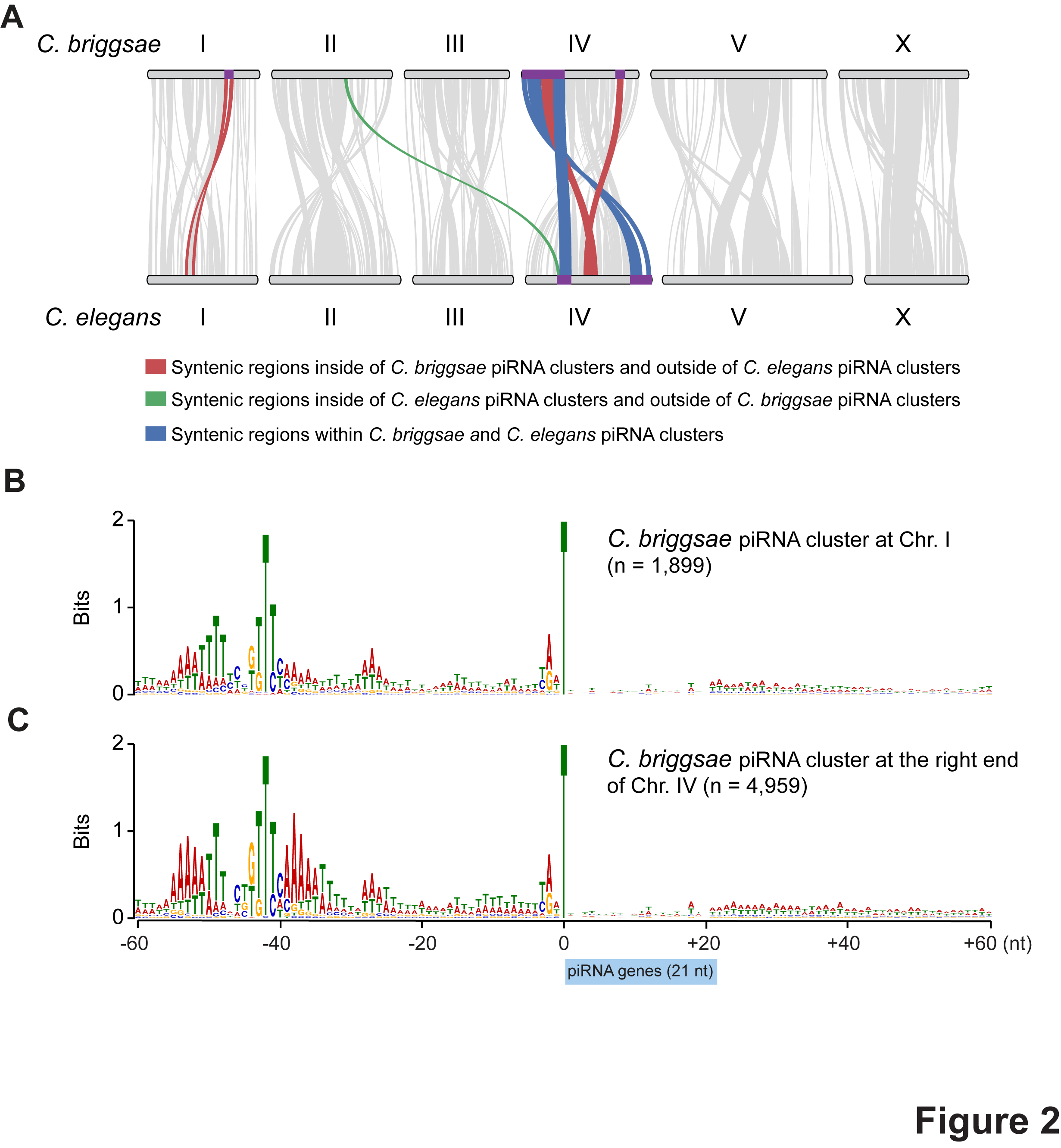

Supplement: Supplemental Material [file KRNB_A_2149170_SM6978.zip › S2.tif]

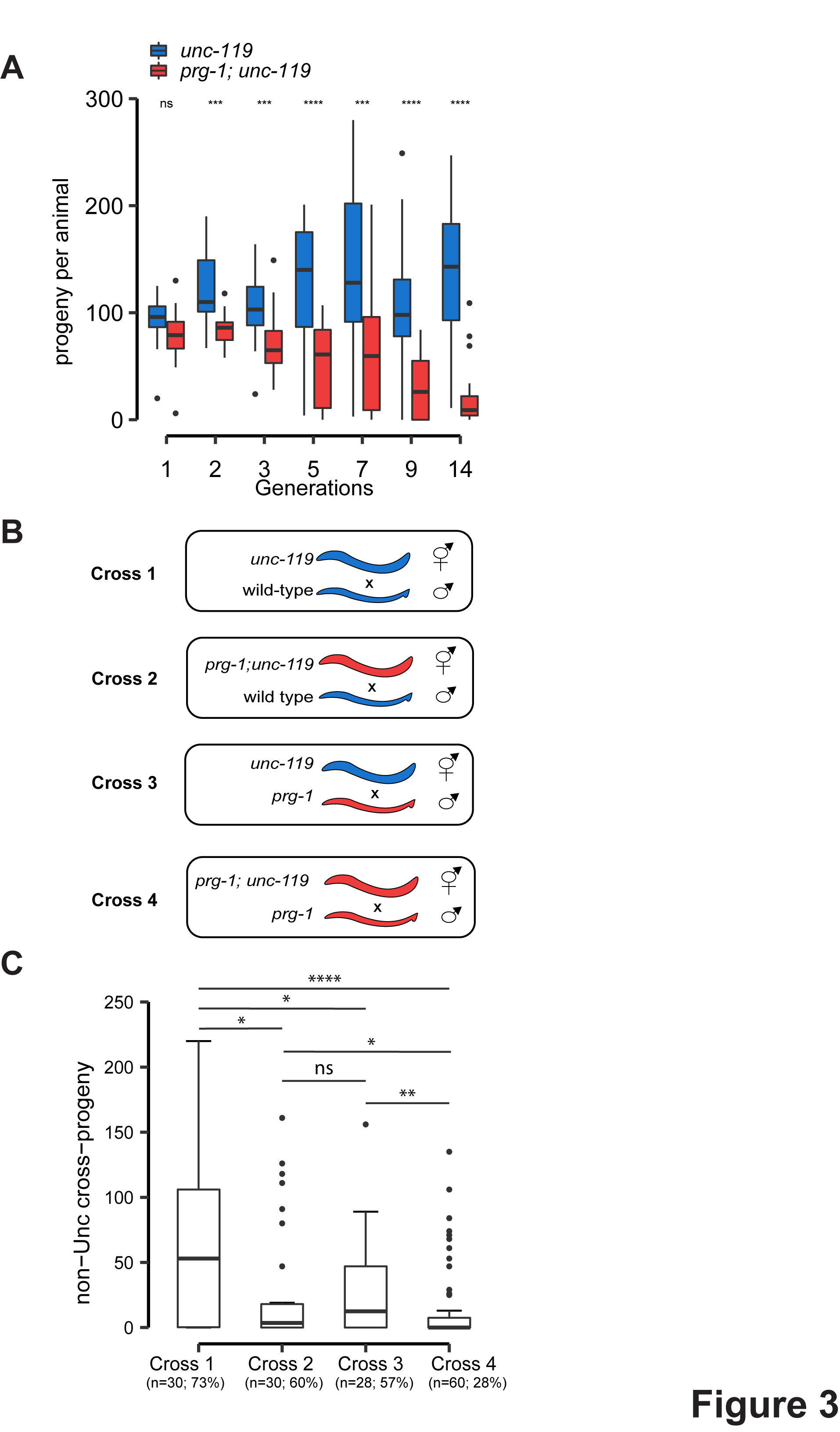

Supplement: Supplemental Material [file KRNB_A_2149170_SM6978.zip › S3.tif]

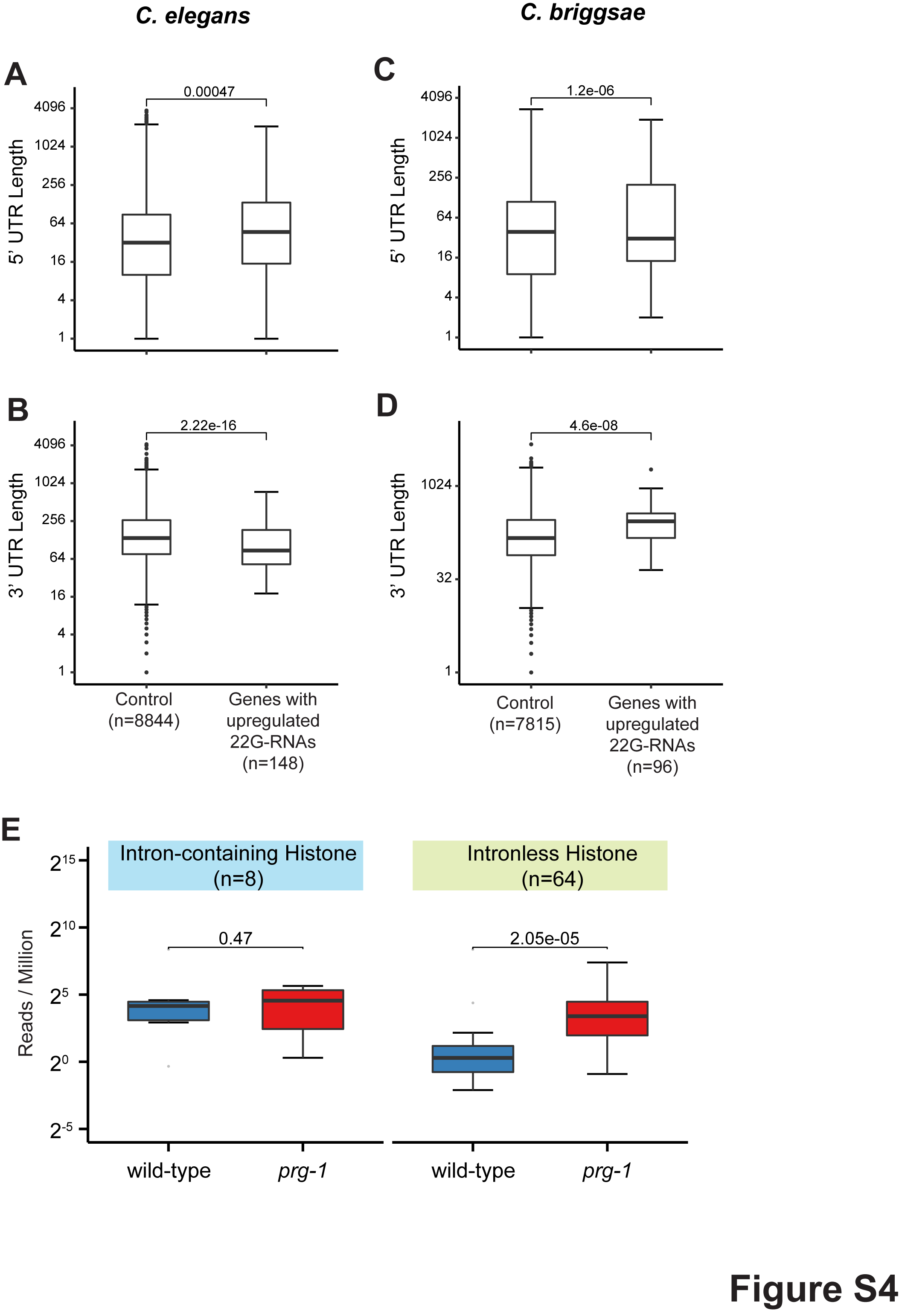

Supplement: Supplemental Material [file KRNB_A_2149170_SM6978.zip › S4.tif]
